# Daniel Frank Whitten, D.M.D.

**Published:** 1891-05

**Authors:** 


### Obituary.

### DANIEL FRANK WHITTEN, D.M.D.

RESOLUTIONS adopted by the Harvard Odontological Society at a regular monthly meeting, held at Young's Hotel, Boston, March 26, upon the death of Daniel Frank Whitten, D.M.D., of South Boston.

WHEREAS, It has pleased Almighty God to remove from among us our friend and associate, Daniel Frank Whitten, D.M.D. Therefore be it

*Resolved*, That in Dr. Whitten's death this Society has lost one of its oldest and most esteemed members and former presidents. Genial in disposition, eminently successful in his profession, Dr. Whitten was one whom we all loved as a man and honored as a dentist.

*Resolved*, That our heart-felt sympathy be extended to the family of the deceased in their bereavement.

*Resolved*, That a copy of these resolutions be spread upon the records of this Society; that a copy be sent to his family, and to the INTERNATIONAL DENTAL JOURNAL for publication.

